# Socio-Demographic and Clinical Correlates of Exclusive Breastfeeding practices up to Six Months of Age

**DOI:** 10.3126/nje.v9i3.21179

**Published:** 2019-09-30

**Authors:** Anil C Mathew, Jibin K Benny, Dona Maria Philip, C Dhanya, Melvin Joy, V Sandhiya, S Ramesh, K Neelakandan

**Affiliations:** 1 Professor of Biostatistics, Department of Community Medicine, PSG Institute of Medical Sciences and Research, Coimbatore 641 004, Tamil Nadu, India.; 2 Trainee Biostatistician, Department of Statistics and Biostatistics, St. Thomas College, Pala, Kerala.; 3 MD Community Medicine First year, Department of Community Medicine, PSG Institute of Medical Sciences and Research, Coimbatore 641 004, Tamil Nadu, India.; 4 Associate Professor of Paediatrics, Department of Paediatrics PSG Institute of Medical Sciences and Research, Coimbatore 641 004, Tamil Nadu, India.; 5 Professor and HOD of Paediatrics, Department of Paediatrics PSG Institute of Medical Sciences and Research, Coimbatore 641 004, Tamil Nadu, India.

**Keywords:** Exclusive breast feeding, socio-economic status, mother’s age

## Abstract

**Background:**

According to the recommendations from The World Health Organization, exclusive breast feeding (EBF) should be given to infants for the first six months for healthy growth and development. However, developing countries have a wide range of variation in the practice of EBF. From this study, we aim to estimate the duration of EBF and find the impact of socio-demographic characteristics on EBF.

**Materials and Methods:**

This study was conducted at PSG Institute of Medical Sciences and Research, Coimbatore in the month of July 2018. Mothers with children aged below 4 years attending the paediatric outpatient department were included in the study. Kaplan-Meier curve analysis and logistic regression analysis were employed.

**Results:**

Our analysis indicates that the prevalence of EBF was 58.4% among infants up to 6 months of age with mean duration of EBF 4.52 months. It was observed that mothers of a younger age (15-24 years) and with lower socio-economic status were negatively associated with the practice of providing EBF to infants up to six months of age.

**Conclusion:**

The findings clearly indicate that the interventions related to EBF up to six months of age should be targeted at women in the lower socio-economic status and at younger ages.

## Introduction

According to the recommendation of the World Health Organization (WHO), exclusive breastfeeding (EBF) should be given to infants for the first six months of life and continued breastfeeding up to two years of age or beyond for the healthy growth and development [1-3]. Recent literatures have suggested exclusive breastfeeding as a cost-effective intervention to reduce infant mortality rate in less developed countries [4, 5]. Globally, it is estimated that the lack of providing exclusive breast feeding to infants in the first 6 months of life results in 1.4 million deaths and 10% of disease in under-five children [6]. On the other hand, poor school performance, reduced productivity and impaired intellectual and social development are reported to be much higher due to the lack of EBF in the first 6 months of life.

In India, a large number of children, nearly 20 million, were not able to receive EBF for the first six months, according to the reports from National Family Health Survey (NFHS) [7]. Over the past several years, India has failed to witness any remarkable achievements in infant feeding practices. Only a small increment was recorded in EBF rates amongst infants in the 0-6 month age group from 41.2% in 1998-99 (NFHS 2) to 46.3% in 2005-2006 (NFHS 3). The mean duration of EBF among last born children is as brief as two months.

In 2016, the Government of India started various initiatives to tackle the health issues among children due to inappropriate breast-feeding practices. The Government of India developed the infant and young child feeding guidelines in 2016 in view of this initiative. However, more studies are needed to explore the factors associated with the low proportion of EBF. The present study aims to estimate the duration of EBF and to find the impact of socio-demographic characteristics influences on EBF practice.

## Methodology

### Study design and participants:

A cross-sectional study was conducted at PSG Institute of Medical Sciences and Research, Coimbatore in the month of July 2018. The mothers of infants less than four years of age, who attended the paediatric outpatient department, were included in the study. The selected mothers were broadly classified into two groups: EBF and non-EBF. Mothers having children aged below 6 months and who were given only breast milk in the 24 hours preceding the survey were considered as EBF and others were in non-EBF group [8].

### Sample size calculation:

In order to estimate the EBF with the precision of 20% of the expected prevalence of 30%, with 95% CI, we require a minimum sample size of 300 for the study.

### Data collection:

Data were collected using direct interview method. Mothers were interviewed about the duration of EBF in months with respect to youngest child. The socio-demographic characteristics and breast-feeding related information were elicited from the selected mothers. The characteristics studied were educational status of the mother, age of the mother, working status of the mother, educational status of father, socio-economic status of the household, child’s gender, gestational age of the child, birth weight of the child, mode of delivery, condition of the child soon after delivery, and counselling for breastfeeding after delivery. The socio-economic status was calculated using Prasad classification with class 1 as the person having per-capita income greater than or equal to Rs.6180, class 2 ranging from Rs.3090-6179, class 3 ranging from Rs.1850-3089, class 4 ranging from Rs.930-1849, and class 5 as person having per-capita income below Rs.930. The classification was done based on the Consumer Price Index (CPI) value of 279 for Coimbatore [9].

### Exclusion criteria:

Babies with congenital anomalies that interfered with breastfeeding were not included in the study.

### Ethical committee approval:

The study was approved by the Institutional Human Ethics Committee, PSG Institute of Medical Sciences and Research, Coimbatore.

### Outcome variable:

The outcome variable was the exclusive breast feeding (EBF) status of infants up to six months of age.

### Explanatory variable:

Mother’s age, educational status of mother and father, working status of mother, gender, gestational age and birth weight of the child and socio-economic status of the household were the explanatory variables.

### Data management and statistical analysis:

To estimate the mean and median duration of exclusive breast feeding among infants under study, the Kaplan-Meier Survival analysis method was applied and conditional probability of survival at monthly interval of time was then obtained. The termination of EBF was considered as outcome of the event of the occurrence. Accordingly, those women who fed their babies exclusively with breast feeding on the date of interview were considered as censored cases. In such cases, the duration of EBF was recorded as it was not known when they would discontinue EBF and start introducing adult diet. The duration of exclusive breastfeeding was sub grouped into smaller time intervals and for each interval all those who have been observed at least that long were used to calculate the probability of a terminal event occurrence in the interval. Then the overall probability of the event occurrence at different time periods were estimated using the probabilities estimated from each interval. Survival probabilities, standard error, hazard rate, and mean and median survival time have been calculated. Log rank test was applied to compare the statistical significance for the equality of duration of exclusive breast feeding for different groups. In addition, logistic regression analysis was done to find the association of socio-demographic characteristics with EBF categorized as Yes or No. Odds ratio and then 95% confidence intervals were then calculated. Multivariate logistic regression analysis was done for those variables found significantly associated with the outcome variable of interest in the Univariate analysis. p<0.05 was considered as statistically significant. The data were analysed using SPSS (Ver. 23).

## Results

A total of 527 women were studied. The socio-demographic characteristics of infants and families are given in Table 1. The prevalence of exclusive breast feeding was 58.4% among infants up to 6 months of age with the mean duration of EBF as 4.52 months.

On testing equality of the duration of EBF, it was observed that the mean duration of EBF was higher in mothers of age group 35-44 years than those aged 15-24 years and 25-34 years (p<0.05). The mean duration of EBF was more in class 1 and 2 of the socio-economic status than class 3,4, and 5 of the socio-economic status (p<0.05). The mean duration of EBF was more in children whose father’s education was above 12th standard than those less than 12th standard (p<0.05). It was also observed that the mothers with the sick children tend to do less EBF than those with well children (p<0.05). The findings are presented in Table 2.

In the logistic regression analysis (Table 3), the prevalence of EBF was higher among the mothers of age group 25-34 years than those aged 15-24 years (p<0.05) and it was significantly higher among class 1 and 2 of the socio-economic status than class 3, 4, and 5 of socio-economic status (p<0.01). The multivariate logistic regression analysis also shows that the socio-economic status of class 1 and 2 had significantly higher prevalence of exclusive breast feeding than class 3, 4, and 5 of the socio-economic status (p<0.01) with an odds ratio of 1.788 (95% CI: 1.153-2.775). In addition, the prevalence of EBF was higher among the age group 25-34 years than in the aged group 15-24 years (p<0.05) with an odds ratio of 1.572 (95% CI: 1.021-2.421).

During the interviews, 47 (8.9%) mothers reported the problem of inverted nipple as a constraint for EBF. Seventy-seven (14.6%) mothers perceived to have the problem of low breast milk and 124 (23.5%) mothers had employment outside home. In addition, 151 (28.6%) mothers had no supportive family members at home (Table 4). It was also observed that there was a statistically significant association between perception of low breast milk and lower socio-economic status (p<0.05) (Table 4).

## Discussion

### Prevalence of exclusive breastfeeding

Breast feeding is universally recognized as the best way of feeding infants which helps in their healthy growth and development. Therefore, several countries, especially developing nations, have started implementing health promotion interventions to support successful breastfeeding by taking into account the factors influencing breast feeding outcomes. For infants till the first six months of life, the WHO strongly recommends EBF without any additional food or water. The WHO also suggests providing complementary diet at 6 months along with breast milk and sustaining breast feeding until the age of 2 years. However, whether an infant is breastfed or formula fed depends on several factors like social, psychological, and environmental scenarios.

Despite several efforts made for EBF up to six months, in our study, 42% of the mothers were still not following EBF up to six months. The mean duration of EBF was 4.52 months which is much lower than the findings reported by Chudsama et al. [5]. Infants not exclusively breast fed up to six months are at risk of higher occurrence of associated infections, malnutrition deficiencies, and under-development [10, 11]. Most of the recent studies regarding EBF come out with the similar fact that the practice of EBF tends to decline and highlight the tremendous progress in the marketing of industrial milk products as well as the lack of awareness of this serious issue among mothers.

### Socio-economic status and its association with exclusive breast feeding

A significantly lower prevalence of EBF was observed among mothers with lower socio-economic status, which is controversial with the result obtained from an Ethiopian demographic health survey that explained a declining trend of EBF with the higher maternal educational and socio-economic status [12]. Furthermore, our study found that the duration of EBF was significantly lower among mothers with lower socio-economic status. The findings of our study clearly acknowledge socio-economic status of the household as a major influencing factor for longer breastfeeding practice among mothers in a higher income bracket. In addition, we have also observed a significant association between the perception of low breast milk and lower socio-economic status. The findings clearly suggest that interventions should be tailored to target women with lower socio-economic status.

### Age of the mother and its association with exclusive breast feeding

It was observed that age of the mother is a predictor of EBF. The mean duration of EBF was significantly lower in the age group of mothers with 15-24 years. The findings suggest that more efforts are needed to create awareness about the need for EBF in younger aged mothers.

### Education and occupation of the parent’s and its association with exclusive breast feeding

We could not find statistical significance between the father’s education and increased prevalence of EBF in the multivariate analysis though we observed a statistical significance in the univariate analysis. Maternal educational status was also found statistically not significant with EBF, which is a controversial observation with the findings of the Ethiopian demographic health survey which indicated a declining trend of EBF practices with higher maternal educational status but supports the findings observed by Tesfaye Setegn et al. [10].

## Conclusion

The findings clearly indicate that the interventions related to EBF up to six months of age should be targeted to women in the lower socio-economic status and at younger ages.

### Limitation of the study:

Our study relies on retrospective reporting, which may result in inaccurate recall and this can be point out as a major limitation. However, most of the recent literature also followed the similar maternal recall mode for elucidating breast-feeding practices. Since this was a hospital-based study, the study population was representative of generally higher socio-economic status and which also reflects as a limitation of our work. A community-based cross-sectional study with a large sample size can provide more light in this field. Despite these limitations, the study has several strengths. We have elicited a large number of demographic and clinical data which can influence the association of duration of EBF. The same interviewer collected all information which reduces the inter observer bias. Advanced statistical methods were employed to analyse the association of socio-demographic and clinical correlates with EBF.

### Future scope of the study:

Our results can help to create more awareness about the need for EBF in the younger age group and the mothers in lower socio-economic status.

### What is already known on this topic?

To the best of our knowledge, not many studies were conducted in Tamil Nadu to examine the socio-demographic and clinical correlates of exclusive breastfeeding. Results of this study indicate a need to create awareness to mothers regarding the need of providing EBF to infants up to six months of age.

### What this study adds:

India has undergone a rapid transition. Contradictory to our expectations, higher socio-economic status mothers are now aware about the need for EBF. More efforts are needed for those mothers in lower socio-economic status and those at younger ages. Secondly, apart from mother’s education, father’s education also plays a great role in breast feeding practices. In conclusion, it was observed that lower socio-economic status and young age of the mother have negative impact on EBF for six months of age.

## Figures and Tables

**Table 1:** Socio demographic and clinical characteristics of children and Families

Characteristics	Number (%)
Child’s age (months) (Mean±SD)	15.55 ± 11.92
**Education of the Mother**	
Below 12th standard	138 (26.2)
Above 12th standard	389 (73.8)
**Mother’s age (years)**	
15-24	108 (20.5)
25-34	388 (73.6)
35-44	31 (5.9)
**Mother’s Occupation**	
House wife	403 (76.5)
Paid worker	124 (23.5)
**Education of the Father**	
Below 12^th^ standard	129 (24.5)
Above 12^th^ standard	398 (75.5)
**Socio-economic status**	
Class 1 and 2	426 (80.8)
Class 3,4 and 5	101 (19.2)
**Gender of the child**	
Female	246 (46.7)
Male	281 (53.3)
**Gestational age of the child**	
Term	469 (89.0)
Preterm	58 (11.0)
**Birth weight of the child (gm)**	
<=2500	126 (23.9)
>2500	401 (76.1)
**Mode of delivery**	
Vaginal delivery	336 (63.8)
Caesarean	191 (36.2)
**Condition of the child**	
Well	454 (86.1)
Sick	73 (13.9)
**Counselling after delivery**	
Yes	326 (61.9)
No	201 (38.1)

**Table-2:** Duration of exclusive breast feeding according to the Socio-demographic characteristics

Characteristics	Mean duration of EBF (months)	Median duration of EBF (months)	^*^Chi-square	p value
**Education of the Mother**				
Below 12^th^ standard	4.33	5.00	0.89	0.345
Above 12^th^ standard	5.57	6.00		
**Mother’s age (years)**				
15-24	4.15	5.00	7.44	P<0.05
25-34	4.55	6.00		
35-44	5.04	6.00		
**Mother’s Occupation**				
House wife	4.49	6.00	0.19	0.663
Paid worker	4.58	6.00		
**Education of the Father**				
Below 12^th^ standard	4.15	5.00	4.49	P<0.05
Above 12^th^ standard	4.62	6.00		
**Socio-economic status**				
Class 1 and 2	4.61	6.00	5.45	P<0.05
Class 3,4 and 5	4.10	5.00		
**Gender of the child**				
Female	4.58	6.00	0.15	0.690
Male	4.44	6.00		
**Gestational age of the child**				
Term	4.58	6.00	3.88	0.143
Preterm	4.02	6.00		
**Birth weight of the child**				
≤2500	4.33	6.00	0.11	0.772
>2500	4.58	6.00		
**Mode of delivery**				
Vaginal delivery	4.58	6.00	0.20	0.654
Caesarean	4.41	6.00		
**Condition of the child**				
Well	4.63	6.00	5.07	P<0.05
Sick	3.84	5.00		
**Counselling after delivery**				
Yes	4.54	6.00	0.05	0.860
No	4.493	6.00		
**Total**	4.523	6.00		

* indicates Log rank Chi square

**Table-3:** The association of Socio demographic characteristics on exclusive breast feeding

Characteristics	EBF: n (%)	Odds Ratio	95% CI	p Value
**Education of the Mother**				
Below 12^th^ standard	76 (55.1)	1		
Above 12^th^ standard	232 (59.6)	1.205	0.815-1.784	0.350
**Mother’s age (years)**				
15-24	53 (49.1)	1		
25-34	235 (60.6)	1.594	1.038-2.447	P<0.05
35-44	20 (76.3)	1.887	0.825-4.313	0.132
**Mother’s Occupation**				
House wife	233 (57.8)	1		
Paid worker	75 (60.5)	1.117	0.741-1.684	0.598
**Education of the Father**				
Below 12^th^ standard	66 (51.2)	1		
Above 12^th^ standard	242 (60.8)	1.481	0.993-2.208	0.054
**Socio-economic status**				
Class 1 and 2	261 (61.3)	1.817	1.174-2.813	P<0.01
Class 3,4 and 5	47 (46.5)	1		
**Gender of the child**				
Female	146 (59.3)	1.072	0.758-1.518	0.693
Male	162 (47.4)	1		
**Gestational age of the child**				
Term	275 (58.6)	1		
Pre-term	33 (56.)	0.931	0.537-1.616	0.800
**Birth weight of the child**				
<=2500	72 (57.1)	1		
>2500	236 (58.9)	1.073	0.715-1.608	0.734
**Mode of delivery**				
Vaginal delivery	202 (60.1)	1		
Caesarean	106 (55.5)	0.827	0.578-1.185	0.301
**Condition of the child**				
Well	271 (59.7)	1		
Sick	37 (50.7)	0.694	0.423-1.139	0.149
**Counselling after delivery**				
Yes	192 (58.9)	1		
No	116 (57.7)	0.952	0.667-1.360	0.789
**Total**	308 (58.4)			

**Table-4:** Association of barriers to exclusive breast feeding with socio-economic status

Constraints	Class 1 and 2: n (%)	Class 3, 4 and 5: n (%)	p value
**Inverted flat nipple**			
Yes	37 (8.7)	10 (9.9)	0.482
No	389 (91.3)	91 (90.1)	
**Perception of low breast milk**			
Yes	56 (13.1)	21 (20.8)	P<0.05
No	370 (86.9)	80 (79.2)	
**Employment outside home**			
Yes	111 (26.1)	13 (12.9)	P<0.01
No	315 (73.9)	88 (87.1)	
**Supportive family members**			
Yes	303 (80.6)	73 (72.3)	0.818
No	123 (19.4)	28 (27.7)	
